# Editorial: Safe utilization of heavy metals pollution in soils for healthy food

**DOI:** 10.3389/fpls.2024.1511483

**Published:** 2024-11-11

**Authors:** Jiawen Wu, Qi Tao, Muhammad Bilal Khan

**Affiliations:** ^1^ College of Life Sciences, Yan’an University, Yan’an, China; ^2^ College of Resources, Sichuan Agricultural University, Chengdu, China; ^3^ Soil and Water Testing Laboratory Layyah, Ayub Agriculture Research Institute, Faisalabad, Pakistan

**Keywords:** alleviation, bioremediation, crop, heavy metal, phytoremediation, detoxification, exogenous supply, inoculation

Heavy metal contamination affects more than five million soil locations globally. Heavy metals can be found in the soil through natural processes or as a result of human activity. Over the past few decades, a number of factors have contributed to the deposition of heavy metals in the global soil system: they include fast industrial growth, air pollution, irrigation using contaminated water, sewage sludge application, excessive pesticide usage, and the overuse of inorganic fertilizers. On the one hand, these toxic heavy metals in the soil damage photosynthesis, respiration, transpiration and other metabolic processes in plants resulting in retardation of plant growth or reduced crop yield. On the other hand, heavy metals enter the human body through the food chain, which leads to kidney diseases, liver diseases, central nervous system disorders, and insomnia. Therefore, it is imperative to develop strategies for the safe utilization of heavy metal-polluted soils. Nevertheless, environmentally-friendly, cost-effective, efficient and sustainable strategies to promote the phytoremediation efficiency of heavy metal-polluted soils or to suppress toxic heavy metal accumulation in edible organs of crops are still limited. Thus, this Research Topic highlights recent developments, current knowledge and perspectives on phytoremediation or mitigation of heavy metal stress in plants for the safe utilization of heavy metal-polluted soils.

A total of two review articles are included in the current Research Topic. Modern techniques for the analysis of heavy metals in plants have been reported by He et al., along with a discussion of their advantages and disadvantages. Hao et al. showed that arbuscular mycorrhizal fungi (AMF) inoculation reduced arsenic (As) accumulation in plants in a dose-dependent manner, and single AMF inoculation reduced As in plants more than mixed inoculation by using a meta-analysis. Since cadmium (Cd) is of utmost significance in polluted soil, the majority of the articles published in this Research Topic focus on the phytoremediation of Cd-polluted soil or the amelioration of Cd stress in plants.

Developments in the phytoremediation of Cd-polluted soils by stimulating Cd accumulation in plants are also presented. According to Wang et al., inoculating maize roots with a single AMF or dark septate endophytic fungus (DSE) significantly increased the concentration of Cd, whereas inoculating maize roots with DSE significantly enhanced the accumulation of Cd in both the shoots and the roots. Significant positive interactions between AMF and DSE on photosynthetic rate (Pn), root glutathione metabolism, and endogenous hormone contents suggest that AMF and DSE inoculation confers Cd tolerance in maize. Hu et al. reported that exogenous silicon (Si) application increased biomass, Cd translocation factor and Cd concentrations in shoots of *Sedum alfredii* Hance, resulting in a significant increase in Cd accumulation in this Cd hyperaccumulating plant. Moreover, Si application elevated chlorophyll content, improved antioxidant capacity, enhanced cell wall components, increased secretion of low molecular weight organic acids, and regulated expression levels of *SaNramp3*, *SaNramp6* and *SaCAD*, which are involved in Cd detoxification. Therefore, Si facilitates the phytoremediation of Cd by *S. alfredii*. Xie et al. elucidated that Cd acidified and improved organic carbon accumulation in the rhizosphere soil of *Siegesbeckia orientalis* L. contributing to active Cd bioavailability. Active organic carbon accumulation further reversed Cd-attenuated microbial and enzymatic activities in the rhizosphere soil. These findings provide new insights for improving the phytoremediation efficiency of the Cd-polluted soil.

The role of exogenous agents in reducing Cd uptake, transport, distribution and accumulation in plants is also explored. Tang et al. demonstrated that applying 100-500 mg L^-1^ salicylic acid (SA) increased lettuce growth and decreased Cd concentrations in lettuce shoots. SA promoted photosynthetic capacity, reduced Cd-induced oxidative stress, and downregulated expression levels of *Nramp5*, *HMA4*, and *SAMT*, which were the reasons for reducing Cd toxicity in lettuce. Hira et al. found that combining biochar application, *Rhizobium leguminosarum* inoculation and *Vigna radiata* intercropping promoted mustard growth rates by 65%–90% in the early growth stage and by 70%–90% in the mature stage. The combination remarkably reduced Cd translocation in mustard plants by 70%–95%. El-Sappah et al. discovered that foliar spraying of silymarin and clove fruit extract acted as a potent novel biostimulator that improved growth, photosynthetic efficiency, non-enzymatic and enzymatic antioxidants, grain yield, and limited Cd accumulation in wheat. Zhuang et al. revealed that higher contents of soil organic matter, cation exchange capacity, clay and silt, and lower sand contents accumulated less Cd in apple trees by enhancing antioxidant activities and downregulating expression levels of genes related to Cd absorption and transport.

Mutual effects of Cd with other environmental factors on plant growth and adaptation are discussed in this Research Topic. Shi et al. showed that original polyethylene microplastics absorbed more Cd from solution, thus increasing Cd accumulation in sweet potato tissues and exacerbating Cd toxicity. Correspondingly, Cd facilitated polyethylene microplastics in the cortex of roots but reduced their accumulation in shoots of sweet potato. Feng et al. demonstrated that a relatively high temperature of 27°C and medium/high CO_2_ levels ameliorated the reduced plant growth of rice caused by Cd and salinity stress. The highest amounts of Cd were found in rice at 27°C and mild CO_2_ levels. The distribution and accumulation of Cd in plants may be affected either positively or negatively by the combined environmental factors.

In summary, inoculation with microbes such as AMF or DSE, enhancement of Cd detoxification capacity by exogenous Si supply, and activation of soil Cd bioavailability by adjusting soil organic carbon are strategies supported by this Research Topic to improve phytoremediation of the Cd-polluted soils. Exogenous application of SA, biochar-*Rhizobium leguminosarum* inoculation-leguminous plants intercropping combination, and silymarin-clove fruit extract compounds are strategies provided by this Research Topic for decreasing Cd contamination in crops. Furthermore, factors such as microplastics, temperature, atmospheric CO_2_ and salinity are worthy of consideration as they commonly co-exist with Cd pollution in the soil.

